# Multiple Risk Factor Intervention Trial Revisited: A New Perspective Based on Nonfatal and Fatal Composite Endpoints, Coronary and Cardiovascular, During the Trial

**DOI:** 10.1161/JAHA.112.003640

**Published:** 2012-10-25

**Authors:** Jeremiah Stamler, James D. Neaton, Jerome D. Cohen, Jeffrey Cutler, Lynn Eberly, Gregory Grandits, Lewis H. Kuller, Judith Ockene, Ronald Prineas, The MRFIT Research Group

**Affiliations:** 1 Northwestern University Medical School, Chicago, IL (J.S.); 2 University of Minnesota, Minneapolis, MN (J.D.N., L.E., G.G.); 3 St. Louis University School of MedicineSt. Louis, MO (J.D.C.); 4 National Heart, Lung, and Blood Institute, National Institutes of Health, Bethesda, MD (J.C.); 5 University of Pittsburgh, Pittsburgh, PA (L.H.K.); 6 University of Massachusetts Medical School, Worcester, MA (J.O.); 7 Wake Forest University School of Medicine, Winston-Salem, NC (R.P.)

**Keywords:** clinical trials, primary prevention, risk factors

## Abstract

**Background:**

The Multiple Risk Factor Intervention Trial evaluated a multifactor intervention on coronary heart disease (CHD) in 12 866 men. A priori defined endpoints (CHD death, CHD death or nonfatal myocardial infarction, cardiovascular disease [CVD] death, and all-cause death) did not differ significantly between the special intervention (SI) and usual care (UC) groups over an average follow-up period of 7 years. Event rates were lower than anticipated, reducing power. Other nonfatal CVD outcomes were prespecified but not considered in composite outcomes comparing SI with UC.

**Methods and Results:**

Post-trial CVD mortality risks associated with nonfatal CVD events occurring during the trial were determined with Cox regression. Nonfatal outcomes associated with >2-fold risk of CVD death over the subsequent 20 years were combined with during-trial deaths to create 2 new composite outcomes. SI/UC hazard ratios and 95% confidence intervals were estimated for each composite outcome. Of 10 during-trial nonfatal events, 6 were associated (*P*<0.001) with >2-fold risk of CVD death. A CHD composite outcome (CHD death, myocardial infarction [clinical or serial ECG change], CHF, or coronary artery surgery) was experienced by 520 SI and 602 UC men (SI/UC hazard ratio = 0.86; 95% confidence interval, 0.76–0.97; *P*=0.01). A CVD composite outcome (CHD [as above], other CVD deaths, stroke, or renal impairment) was experienced by 581 SI and 652 UC men (hazard ratio = 0.89; 95% confidence interval, 0.79–0.99; *P*=0.04).

**Conclusions:**

In post hoc analyses, composite fatal/nonfatal CHD and CVD rates over 7 years were significantly lower for SI than for UC. These findings reinforce recommendations for improved dietary/lifestyle practices, with pharmacological therapy as needed, to prevent and control major CVD risk factors.

## Introduction

Results of the Multiple Risk Factor Intervention Trial (MRFIT) were reported in 1982.^1^ Men assigned to special intervention (SI) had a nonsignificantly lower rate (7.1% lower) of fatal coronary heart disease (CHD), the primary endpoint, than men assigned to usual care (UC). The trial was underpowered as a consequence of fewer deaths having been observed than anticipated, which resulted in a wide confidence interval (CI) for the SI/UC hazard ratio (HR) for fatal CHD after an average follow-up period of 7 years (95% CI, 0.72–1.20). In 1997, when this article was reprinted as a Landmark Report, the accompanying Landmark Perspective commentary underscored this problem of inadequate statistical power and the reasons for it.^2^ Subsequent work confirmed that pretrial exclusions made during screening had a substantial effect on the mortality rates observed during the trial.^3^ Other factors such as secular trends in CHD mortality rates and unanticipated changes made by UC participants likely also contributed to reduced mortality.^1^

To address the loss of power, we here construct, for the first time, a CHD composite outcome and a cardiovascular disease (CVD) composite outcome, which include nonfatal and fatal CVD events, for comparing the SI and UC groups. The nonfatal outcomes were prespecified,^4^ but the composites considered here were not. To inform construction of 2 composite outcomes and the clinical relevance of each, we take advantage of the long-term mortality follow-up of MRFIT participants after closure of the trial.

## Methods

### Study Participants

MRFIT methods have been reported in detail.^1,3–6^ In all, 12 866 men assessed to be in the upper 10% to 15% of CHD risk on the basis of higher levels of serum cholesterol, diastolic blood pressure (BP), and cigarette use were randomized. The UC group (n=6438) was offered no intervention program; they were referred to their usual source of medical care and were examined annually. The SI group (n=6428) participated in an in-depth sustained multifactor intervention program aimed at lowering serum cholesterol and BP and at smoking cessation.^7–10^ Active follow-up of participants and the SI ceased in February 1982. Each participant was followed up for a minimum of 6 years; average follow-up was 7 years.

### Prespecified Clinical Outcomes

A key purpose of annual examinations was ascertainment of interim CVD events.^4^ A resting ECG and laboratory tests were performed as part of a comprehensive physical examination. Hospital records were requested for cardiac diagnoses, and reviewers of these records were blinded to treatment group. Event criteria and numbers experiencing nonfatal CVD events have been reported.^4^ No information on post-trial nonfatal CHD and CVD events is available.

During the trial and through to its conclusion on February 28, 1982, deaths were ascertained by clinical center staff. Cause of death was determined by a committee blinded to treatment group.^1^ CHD death, the primary endpoint, included death from myocardial infarction (MI), sudden death, CHF, and coronary artery surgery. Other CVD deaths included deaths from stroke, hypertension with left ventricular failure, and pulmonary embolus, as well as CVD deaths not classifiable into one of the forgoing categories.^1^ After closure of the trial, post-trial deaths were ascertained by using National Death Index and Social Security Administration files.^11–12^ Causes of these deaths were coded according to death certificates and the *International Classification of Diseases* (*ICD;* 9th revision though 1997, 10th revision afterward). Deaths were classified as CVD on the basis of *ICD-9* (390-459) and *ICD-10* (I00-I99) codes.

Subsequent to the primary trial report,^1^ 3 additional deaths (2 UC and 1 SI) during the trial were identified. Two of these (1 UC, 1 SI) were considered CHD deaths on the basis of the underlying cause of death on the death certificate. One CHD death was coded *ICD-9* 410.0 and the other *ICD-9* 429.2; these deaths were subclassified as MI and sudden death, respectively. The third death was due to stomach cancer (*ICD-9* 150.0).

### Statistical Methods

Cox models, stratified by clinical center (22 centers), were used to estimate HRs for a priori defined endpoints through the end of the trial (February 28, 1982) and through 20 years after the end of the trial (February 28, 2002).^13^ For the former, cause of death based on the review committee's was used when available; for the latter, causes of death were based on death certificate codes.

As a first step in constructing CHD and CVD composites incorporating the nonfatal outcomes, associations of during-trial nonfatal events with 20-year post-trial CVD mortality rates were estimated for men alive at study closure (February 28, 1982). Cox models, stratified by clinical center (22 centers), were used to estimate age-adjusted CVD mortality HRs separately for each nonfatal event.^13^

This information was used to construct 2 composite outcomes (fatal/nonfatal CHD and fatal/nonfatal CVD) for comparing the SI and UC groups during the 7-year follow-up period after randomization in intention-to-treat analyses. Nonfatal events associated with a >2-fold increased risk of CVD death in the 20-year post-trial period were included in the CHD and CVD composite outcomes. SI/UC HRs for composite outcomes were estimated with stratified Cox models, as above. In these analyses, follow-up was censored at trial closure for participants who did not experience an event and at the time of death for those who died from causes not being considered in the outcome (eg, non-CVD causes of death). Nonfatal events ascertained at annual visits were assumed to occur at intervals of 365.25 days after randomization. Logistic regression analyses that ignored event times gave nearly identical results.

Multivariate failure time analyses also were carried out.^14^ Cox models, incorporating multiple events per participant, were used to compute a pooled SI/UC HR for the CHD and CVD composite outcomes. Chi-square tests of homogeneity of HRs (whether the SI/UC HRs are similar for different types of events in the composite) also are presented. Cited *P* values are 2 sided. Analyses were performed in SAS, version 9.2 (SAS Institute, Cary, NC, USA).

## Results

Baseline characteristics and risk factor changes have been reported for the SI and UC groups.^1^ Results also have been reported for the primary endpoint, CHD death, and the 3 other major endpoints through trial closure^1,4^; they are summarized below, with inclusion of 3 additional deaths identified after the primary trial report. There were 116 SI and 125 UC CHD deaths through the closing date (HR=0.93; 95% CI, 0.72–1.20). For CHD death or nonfatal MI, there were 396 SI and 432 UC participants with an event (HR=0.92; 95% CI, 0.80–1.05). Death from any cause occurred in 266 SI and 262 UC men (HR=1.02; 95% CI, 0.86–1.20). These deaths were classified as CVD for 139 SI and 146 UC participants (HR=0.95; 95% CI, 0.76–1.20).

The numbers of deaths and HRs for CHD and CVD death and for all-cause death through February 28, 2002 (20 years after trial closure and an average of 27 years from randomization) were as follows: 885 SI and 942 UC CHD deaths (HR=0.94; 95% CI, 0.86–1.03); 1295 SI and 1332 UC CVD deaths (HR=0.97; 95% 0.90–1.05); and 2713 SI and 2735 UC deaths from any cause (HR=0.99; 95% CI, 0.94–1.04).

### Associations of During-Trial Nonfatal CVD Events With Post-Trial CVD Death

**Figure 1.**
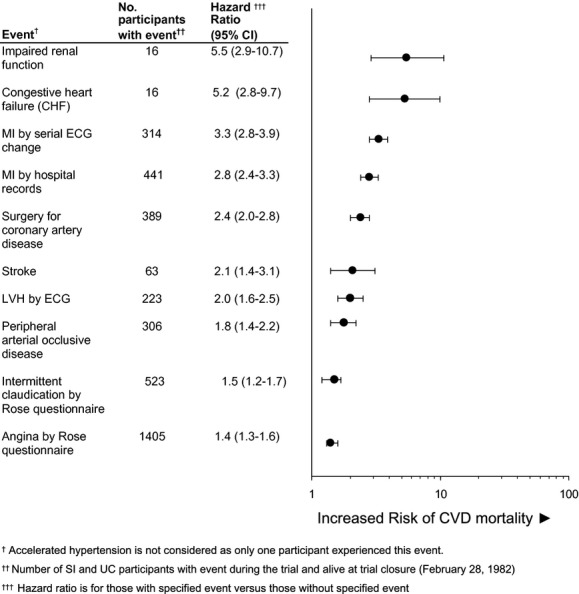
Among participants alive at the end of the trial, number with indicated nonfatal event during the trial and age-adjusted hazard ratios for 20-year post-trial CVD deaths (2340 CVD deaths through February 28, 2002) associated with nonfatal CVD events during the trial (events ranked by hazard ratio). CVD indicates cardiovascular disease; CHF, congestive heart failure; MI, myocardial infarction; and LVH, left ventricular hypertrophy.

Table 1 summarizes the numbers of participants who experienced a nonfatal event during the trial by type of event and by treatment group. For each of these during-trial nonfatal events, we assessed 20-year risk of CVD death (2445 CVD deaths) for the 12 338 participants alive at trial closure (Figure 1). Six events were associated with an HR >2.0 for post-trial CVD death. With serial ECG change and MI by hospital records combined into a single event, HR was 2.9 (95% CI, 2.6–3.4). The HRs for impaired renal function (5.6), CHF (5.0), nonfatal MI by ECG or hospital records (2.6), and stroke (1.9) were similar when the events associated with the greatest risk of CVD death were considered together in a single model with age. The HR for surgery for coronary artery disease declined to 1.7 (95% CI, 1.5–2.1). All remained highly significant (*P*<0.001).

**Table 1.**
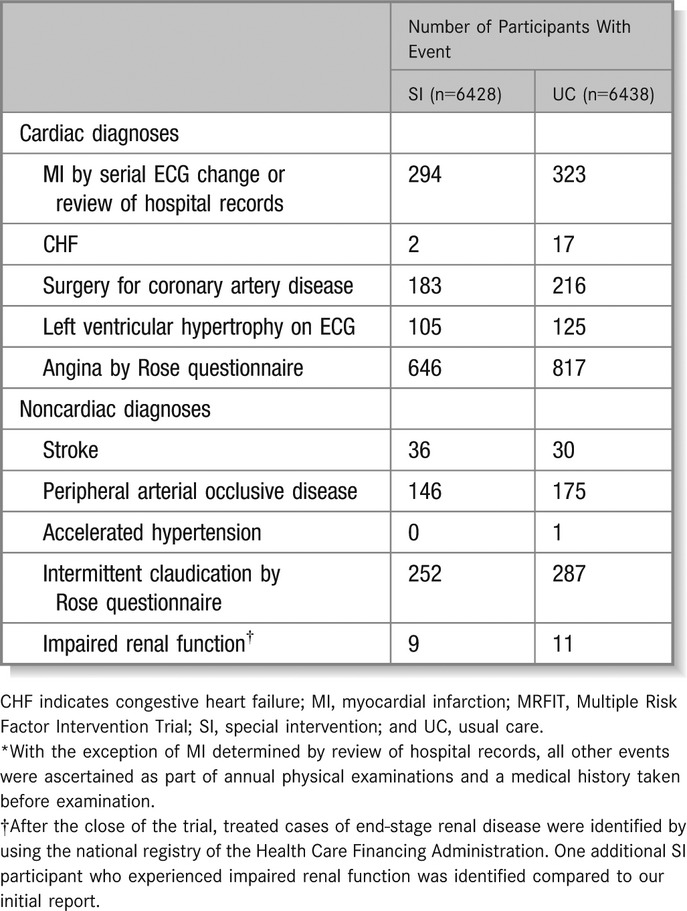
Number of SI and UC Men Experiencing Each Prespecified Nonfatal Cardiovascular Event^*^ Through MRFIT Study Closure (February 28, 1982)

On the basis of these results, 2 composite outcomes were constructed. One includes fatal CHD, nonfatal MI, CHF, and surgery for coronary artery disease. We refer to this as the “CHD composite.” The second composite outcome includes fatal CVD, nonfatal MI, CHF, impaired renal function, surgery for coronary artery disease, and stroke. We refer to this as the “CVD composite.” In each composite outcome, nonfatal events associated with >2-fold increased risk of CVD death when considered singly during the 20-year post-trial period are included as components of the endpoint.

### Comparison of SI and UC Groups for CHD Composite and Components: Outcomes During the Trial (Through February 28, 1982)

Table 2 summarizes findings for the CHD composite outcome. Overall, risk of the CHD composite outcome was 14% lower (*P*=0.01) for SI than for UC. The CHD composite then was broken into 3 separate smaller fatal/nonfatal composites: fatal or nonfatal MI (327 SI and 355 UC participants), fatal or nonfatal CHF (3 SI and 20 UC), and fatal or nonfatal coronary artery surgery (187 SI and 220 UC). These components of the overall CHD composite are not mutually exclusive (ie, some participants experienced >1 type of event and are counted for each). HRs for these 3 outcomes were 0.92 (95% CI, 0.79–1.07; *P*=0.28), 0.15 (95% CI, 0.04–0.50; *P*=0.002), and 0.85 (95% CI, 0.70–1.03; *P*=0.10). In a multiple-events analysis incorporating sudden death along with these 3 CHD outcomes (all with boldfaced lines in the lower half of Table 2) (590 SI and 676 UC CHD events), the pooled SI/UC HR was 0.89 (95% CI, 0.78–1.00; *P*=0.05). SI/UC HRs across these 4 outcomes varied (χ^2^ test *P*=0.03), largely a result of the more extreme HR for CHF. Most CHF events occurred among participants who had been hypertensive at randomization (62% of total) (2 SI and 14 UC).

**Table 2.**
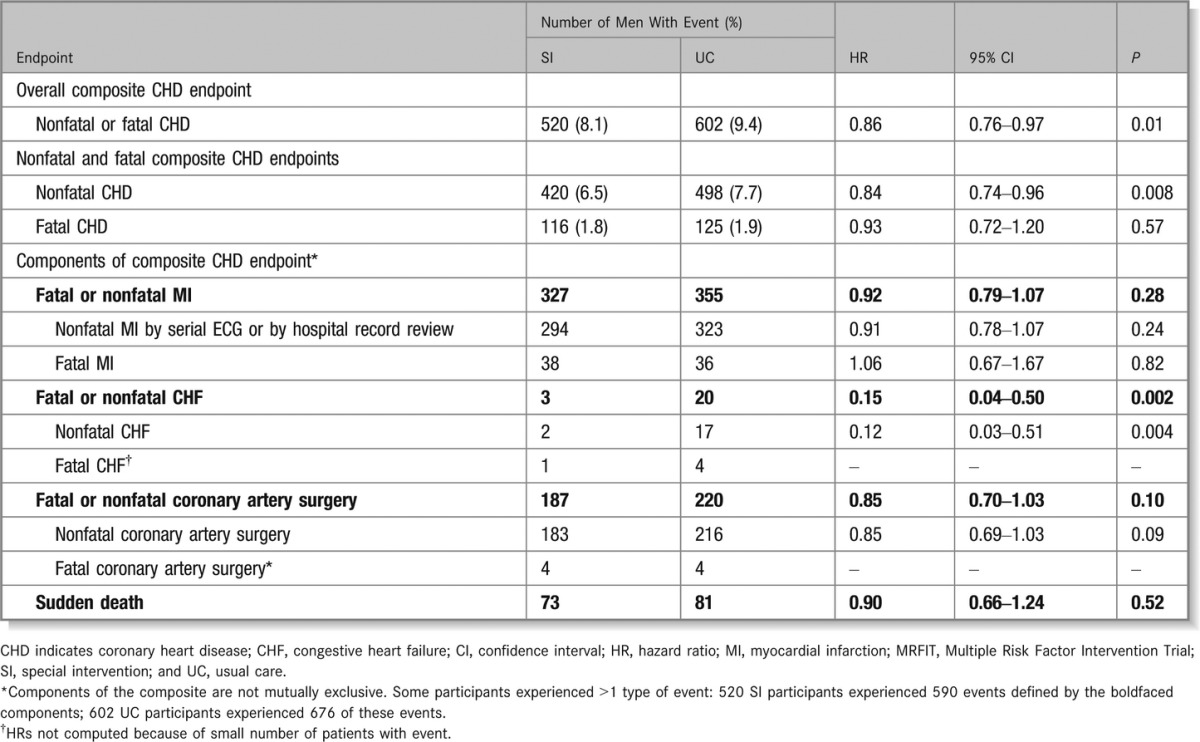
Composite CHD Endpoint: Nonfatal or Fatal CHD Events for SI and UC Participants Through MRFIT Study Closure (February 28, 1982)

We also considered 2 modifications of our composite CHD outcome. We first modified it by including ECG left ventricular hypertrophy (a during-trial event with HR=2.0 for post-trial CVD death), previously shown to be significantly reduced in SI compared to UC hypertensive men.^15^ The SI/UC HR for the first event analysis of this expanded CHD composite was 0.86 (95% CI, 0.77–0.969; *P*=0.005). The second modification excluded CHF to assess sensitivity of the overall results to this component, which strongly favored SI over UC. This resulted in an SI/UC HR of 0.88 (95% CI, 0.78–0.99; *P*=0.03).

### Comparison of SI and UC Groups for the CVD Composite and Components: Outcomes During the Trial (Through February 28, 1982)

Risk of the CVD composite outcome during the trial was 11% lower (HR=0.89, 95% CI, 0.79–0.99; *P*=0.04) for SI than for UC (Table 3). The HR was nearly identical when restricted to men with hypertension at entry (HR=0.88; 95% CI, 0.77–1.01; *P*=0.07). There were 49 SI and 41 UC participants who experienced stroke (HR=1.20; 95% CI, 0.75–1.81; *P*=0.40). Most strokes occurred among hypertensive men (40 SI and 34 UC) (HR=1.17; 95% CI, 0.74–1.85; *P*=0.50).

**Table 3.**
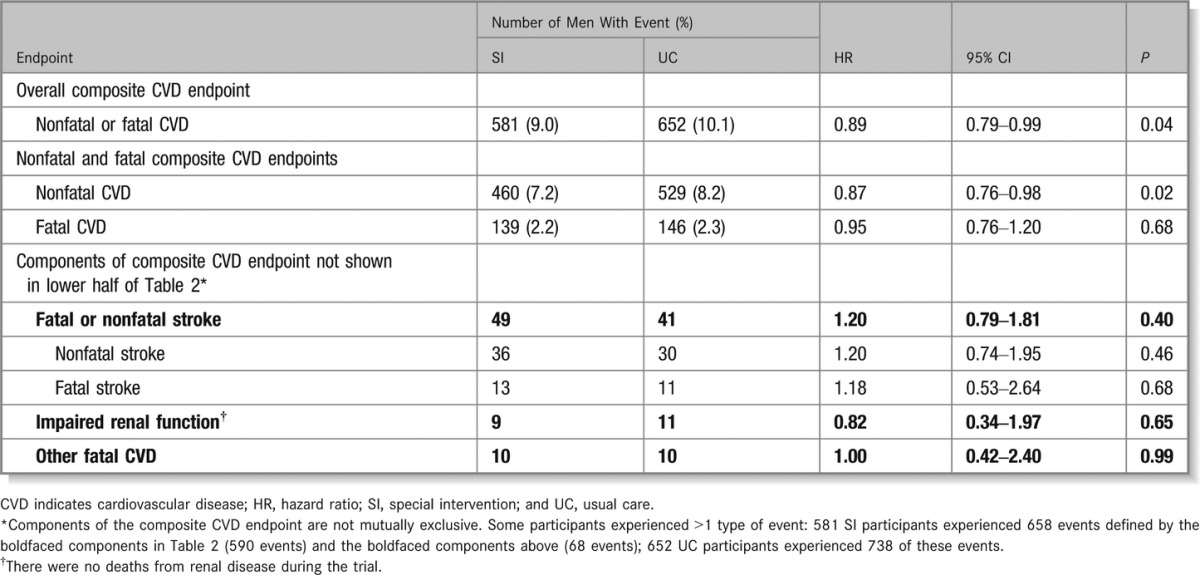
Composite CVD Endpoint: Nonfatal or Fatal CVD Events for SI and UC Participants Through Study Closure (February 28, 1982)

In a multiple-events analysis (the 4 CHD outcomes boldfaced in the lower half of Table 2, plus stroke, impaired renal function, and other CVD death, shown in the lower half of Table 3), 658 events were experienced by 581 SI participants, and 738 events were experienced by 652 UC participants. The pooled SI/UC HR was 0.91 (95% CI, 0.81–1.02; *P*=0.09), and the *P* value assessing homogeneity of SI/UC HRs was 0.10.

## Discussion

Our main finding is that during the active intervention phase of the trial, the SI group had 7-year rates for the composite outcomes of fatal or nonfatal CHD and fatal or nonfatal CVD that were significantly lower than the UC group by 14% (95% CI, 3–24%) and 11% (95% CI, 1–21%), respectively. Analyses that consider multiple events for each participant, not just the first event, are consistent. These analyses also suggest heterogeneity of the effect of intervention on different outcomes. There was a striking benefit of the SI on CHF and modest benefit for other outcomes, with the exception of stroke, for which more SI than UC men had an event.

The significantly lower composite CHD incidence for SI than for UC contrasts with the previously reported nonsignificant difference for the primary endpoint CHD death.^1^ We attribute this in large part to the improved power that resulted from using the composite outcome (ie, 241 CHD decedents versus 1122 participants with CHD composite). The consequence of the larger number of CHD events was both a reduction in the log_e_ HR (from −0.073 to −0.151) and a reduction in the CI width (0.51 to 0.24 after log_e_ transformation). Although the CHD composite considered here was defined post hoc, the components were prespecified during the trial design, and >70% of participants with events had a CHD death or nonfatal MI event that was a prespecified major outcome. The nonfatal CHF and coronary artery surgery components of the CHD composite were associated with 5.2-fold (95% CI, 2.8–9.7) and 2.4-fold (95% CI, 2.0–2.8) increased 20-year risks of death. Furthermore, fatal events attributed to these 2 components were part of the a priori defined CHD death primary endpoint. Thus, their inclusion in the CHD composite, though post hoc, is logical on 2 counts.

Likewise, the inclusion of nonfatal stroke along with fatal stroke in the CVD composite is a logical extension of the CVD death outcome. Impaired renal function, which occurred in only 20 participants, was included in the CVD composite outcome because of the high 20-year risk of death associated with it.

The treatment difference favoring SI for the CHD and CVD composite outcomes during the 7-year trial period did not result in significant treatment differences after 27 years of mortality follow-up. However, there was no planned intervention during the 20 years after the close of the trial, and neither risk factor levels nor incidence of nonfatal events were assessed. Thus, we cannot assess the extent to which risk factor differences were maintained and whether during-trial differences in nonfatal events persisted.

With these considerations, the data on during-trial differences for the post hoc defined CHD and CVD composites reported here indicate that the multifactor intervention program—to achieve sustained smoking cessation and lower elevated serum cholesterol, weight, and BP by dietary/lifestyle means, supplemented as indicated by antihypertensive medication—was effective in preventing clinically relevant CVD events. This was the case even though SI–UC differences in major risk factors were less than expected during the trial period.^1^

The contrasting findings for CHF and stroke are unexpected. In an overview of cohort studies, both stroke and CHF were strongly related to elevated BP^16^: Among men 40 to 49 years of age, a 5–mm Hg lower systolic BP, as observed between SI and UC participants,^1^ would be expected to result in a 24% reduction in deaths from stroke and a 15% reduction in deaths from heart failure.^16^ These differences in risk associated with BP differences accurately predicted results of a recent meta-analysis of BP-lowering trials.^17^

With regard to stroke, we have shown that among the men screened for MRFIT, cigarette smoking is an important risk factor for death from ischemic and hemorrhagic stroke and that risks associated with BP and smoking are additive.^18^ Thus, the predicted reduction in stroke risk for SI men in MRFIT is even greater than 24%.

It is possible that the stroke and CHF findings are due to chance. For both outcomes the number of participants with an event was small. Arguing against this, and in favor of the possibility of a less-than-optimal hypertension intervention program in MRFIT (as previously reported),^19^ are the findings for the men who were hypertensive at baseline, among whom the BP differences between SI and UC at 6 years were 7/5 mm Hg for systolic/diastolic BP. For this subgroup, a very modest, nonsignificantly 12% (95% CI, −1 to 23%) lower CVD composite outcome was observed for SI compared to UC.

Strengths of this analysis include prespecification of several nonfatal outcomes for which post-trial mortality rate could be used for rank-ordering. Other strengths are completeness of follow-up in both the SI and UC groups,^4^ which lessens concerns about differential ascertainment of nonfatal events; ability to assess long-term risk of death associated with different nonfatal events, which allows assessment of the importance of each component; and ability to assess all events a participant developed during the trial, not just the first. MRFIT was not designed as an “event-driven” study, with follow-up continuing until a target number of primary events had occurred. Instead, MRFIT was designed to continue until all participants had been followed up for at least 6 years. Assumptions about key design parameters were incorrect, and this had an adverse effect on power. This analysis aims to overcome that limitation by using clinically relevant composite outcomes that occurred with much greater incidence than the primary endpoint, CHD death.

There are several limitations to our new findings here: First, as noted above, the composite CHD and CVD outcomes were not a priori defined. Also, composites are difficult to interpret if components go in opposite directions.^20^ For the CVD composite, the HR was significantly <1.0 even with inclusion of nonfatal stroke, for which the rate was higher in SI than UC. Secondly, MRFIT was a nonblinded trial. Thus, there is a greater risk of bias in ascertainment of endpoints, particularly nonfatal outcomes. In fact, the original endpoint considered for MRFIT, CHD death or nonfatal MI, was changed to CHD death before beginning the study because of concerns about differential ascertainment.^6^ Finally, these findings pertain to middle-aged men.

In summary, these new overall findings demonstrate that the SI program achieved significantly favorable reductions in CHD and CVD composite outcomes in middle-aged men at above-average risk of CHD. The findings support recommendations, repeatedly made to the public by expert groups, for improved dietary/lifestyle practices (plus pharmacological treatment as needed) to prevent and control established major CHD/CVD risk factors (dyslipidemia, hypertension, diabetes, overweight/obesity, related adverse eating practices, and smoking).
